# Malignant Hemispheric Stroke Secondary to Cavernous Internal Carotid Artery Stenosis in the Setting of Sepsis: A Case Report

**DOI:** 10.7759/cureus.106104

**Published:** 2026-03-30

**Authors:** Natasha Hastings, Amirhossein Zangiabadi

**Affiliations:** 1 School of Medicine, St. George's University School of Medicine, St. George's, GRD; 2 Department of Stroke and Neurology, St. Francis Medical Center, Lynwood, USA

**Keywords:** bladder outlet obstruction, brain death diagnosis, internal carotid artery (ica), report of a rare case, stroke recognition

## Abstract

This is a novel case of a rapidly progressive right hemisphere stroke in the setting of multiple infections that highlights the impact of comorbidities on stroke progression. We hope that reporting this case draws attention to the need for a multidisciplinary focus on the progression of stroke symptoms in the face of septic processes. A 60-year-old African American man with a past medical history of hypertension, diabetes, bilateral above-the-knee amputations, and frequent cocaine use presented to the hospital with left-sided deficits and suspected seizures. The patient’s presentation was complicated by a urinary tract infection (UTI), bladder outlet obstruction, pneumonia, and medication non-compliance. Computed tomography angiography (CTA) revealed severe stenosis of the cavernous segment of the right internal carotid artery (ICA). Magnetic resonance imaging (MRI) revealed a complete right hemisphere stroke with 19.28 mm midline shift. Criteria for brain death were met on day 6, and the patient died on day 10. This case highlights the rapid progression of infarction in the setting of critical internal carotid artery (ICA) stenosis, sepsis, and delayed urinary decompression. Early recognition and coordinated multidisciplinary care may improve outcomes in similarly complex presentations.

## Introduction

Existing literature reports intracranial internal carotid artery (ICA) narrowing (stenosis) much less frequently than extracranial ICA stenosis [1]. Acute ischemic stroke ipsilateral to ICA stenosis (50%-99%) has been reported to occur with ≥70% stenosis [2]. Thrombosis, defined as the formation of a blood clot (thrombus) within a blood vessel, can jeopardize vascular flow. Thrombosis involving the cavernous sinus is a rare and life-threatening disorder with multiple etiologies. Sepsis occurs when the body’s response to infection becomes dysregulated, triggering widespread inflammation and blood flow abnormalities. Complications that arise from underlying septic conditions, or from aseptic causes such as trauma or surgery, and associated risk factors include infection, thrombophilia, and immunosuppression [3]. Infection can indirectly trigger vascular or neurological injury through these inflammatory and coagulation cascades. This case report draws attention to the recognition of stroke progression in the setting of septic disease processes.

## Case presentation

**Figure 1:**
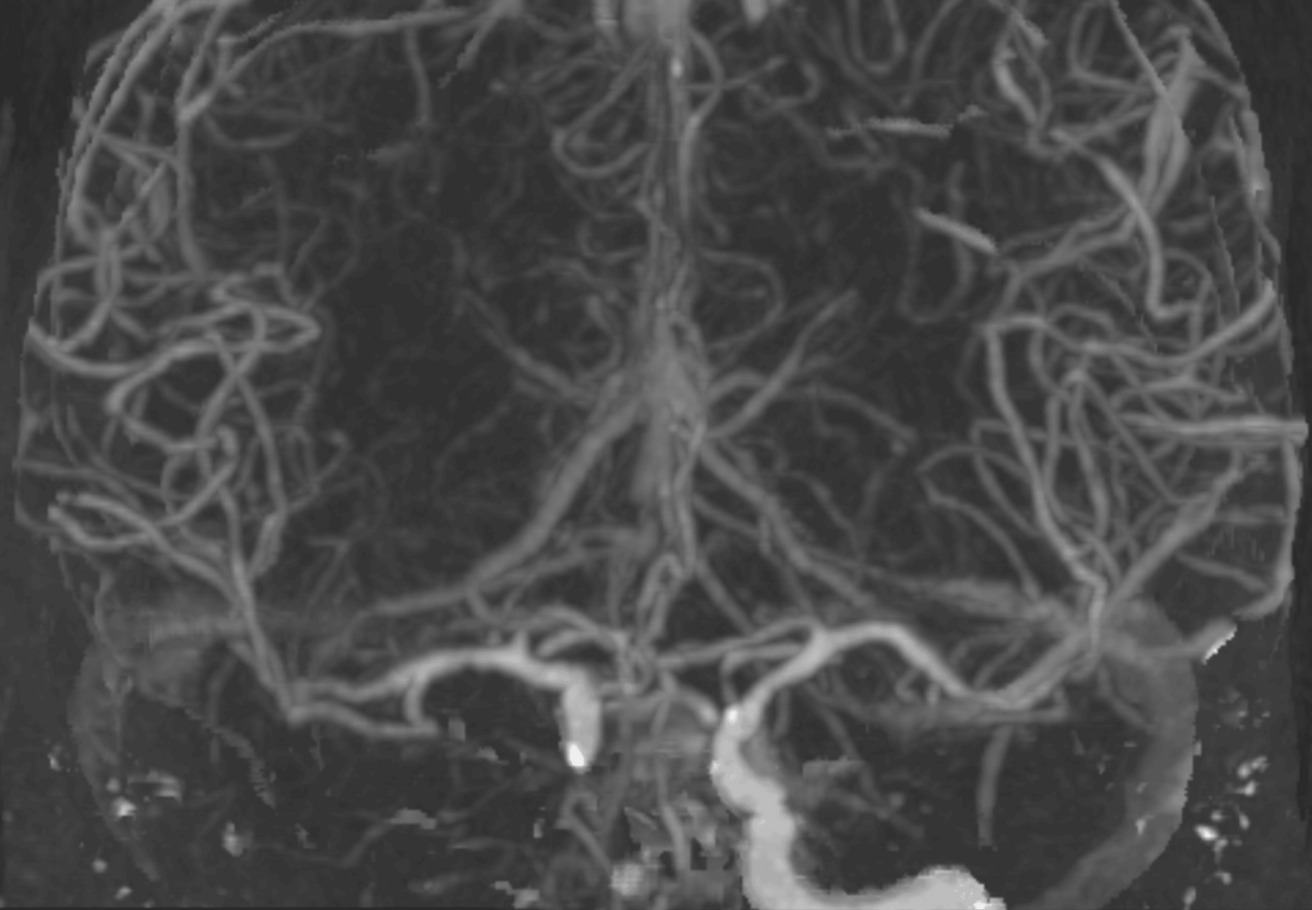
MIP from a CT angiogram of the head demonstrating the intracranial arterial circulation showing severe intracranial stenosis of the right internal carotid artery at the cavernous segment CT: computed tomography, MIP: maximum intensity projection

**Figure 2:**
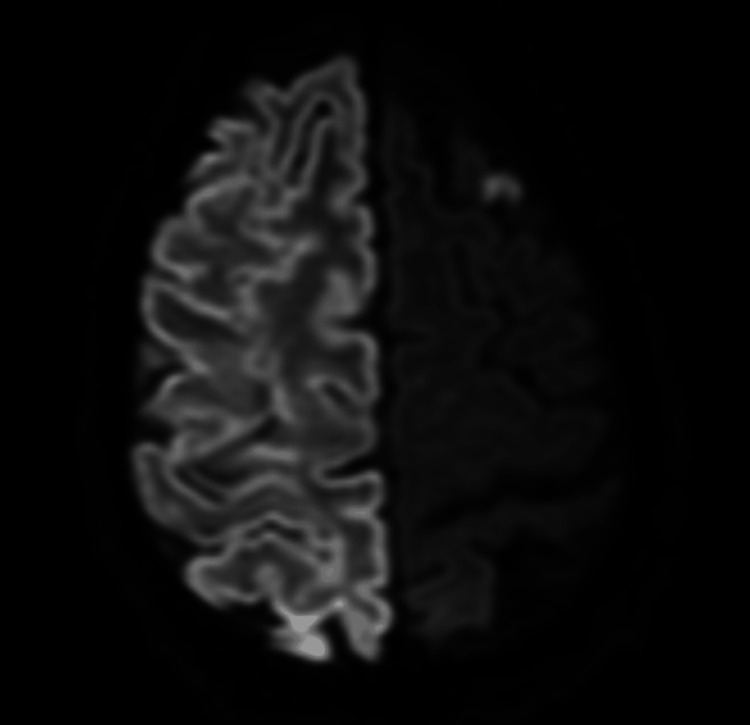
Brain MRI without contrast with diffusion-weighted imaging displaying lack of activity in the right hemisphere post-stroke MRI: magnetic resonance imaging

**Figure 3:**
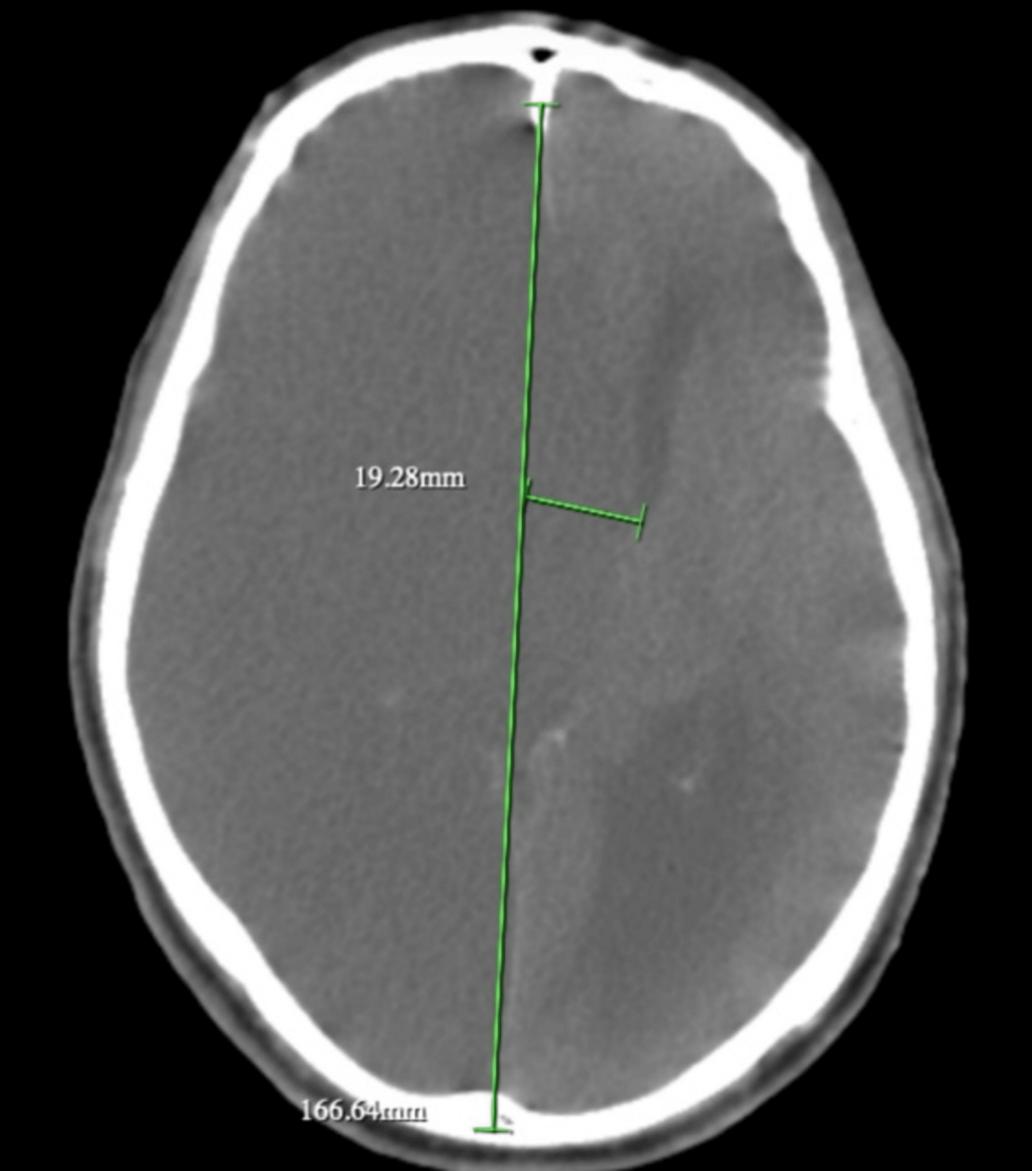
Head CT without contrast displaying 19.28 mm midline shift in the left hemisphere CT: computed tomography

A 60-year-old African American man with a history of hypertension, diabetes, bilateral above-the-knee amputations, frequent cocaine use, and medication non-compliance was brought in by ambulance as a code stroke with suspected seizures. The patient presented with a left-sided facial droop, decreased left-hand grip strength, and pinpoint pupils. Concurrently, he was also suffering from pneumonia and a urinary tract infection (UTI) due to bladder obstruction complicated by colostomy. The patient’s computed tomography angiography (CTA) revealed severe right ICA stenosis, and he was managed with 81 mg aspirin and 80 mg atorvastatin (Lipitor) once daily by mouth for secondary stroke prevention (Figure 1). Stenting was discussed but not indicated at this time due to sepsis (uptrending lactic acid) and renal failure. Additional medications included 500 mg levetiracetam (Keppra) intravenously (IV) twice daily for seizure prophylaxis, 1 g meropenem (Merrem) IV twice daily for broad-spectrum antibiotic coverage, and 40 mg methylprednisolone (Medrol) IV twice daily to control cerebral edema. On day 1, bladder evacuation was delayed, and on day 3, the patient deteriorated overnight with severe acidosis and hypotension requiring intubation. The patient then underwent suprapubic cystostomy placement, and the bladder was irrigated on day 3. Sputum cultures showed light growth of *Escherichia coli *with probable acquired extended-spectrum beta-lactamase (ESBL), and urine cultures grew >100,000 CFU/mL *Proteus mirabilis*, as well as >100,000 CFU/mL *Streptococcus pneumoniae*. On day 4, the patient was not waking up, and a follow-up magnetic resonance imaging (MRI) revealed a massive right-sided stroke involving the entire hemisphere with no evidence of cerebral blood flow, and the patient had lost brain stem reflexes (Figure 2). On day 6, repeat head CT showed evidence of large areas of herniation with midline shift reaching 19.28 mm (Figure 3). A nuclear brain scan showed no evidence of cerebral blood flow. The determination of brain death was reached, and the patient was declared dead on day 10.

## Discussion

Stroke poses a significant burden, with an estimated 16 million new strokes each year and a prevalence exceeding 60 million [4]. Intracranial atherosclerotic disease is a major predictor for ischemic strokes, with middle cerebral arteries being most affected, followed by the basilar artery, ICAs, and vertebral arteries [5]. Our patient exhibited primary signs of a stroke after a history of confounding modifiable risk factors. Concurrent history of cocaine use further amplified the patient’s susceptibility to stroke due to accelerated atherosclerosis and vasospasm. Presentation was acute, and medical management was unsuccessful in combating his poor prognosis. His septic state nullified the opportunity to intervene surgically, and sepsis itself can precipitate cerebral hypoperfusion and microvascular thrombosis.

This case highlights the potential interaction between systemic inflammatory stressors and severe intracranial stenosis. Systemic infections can trigger acute stroke in patients with vascular risk factors due to the inflammatory mediators that contribute to cerebral arteriopathy [6]. This is also known to work retrospectively, where stroke predisposes to infectious complications. Proposed mechanisms of stroke pathogenesis in connection with infections detail direct invasion of arterial walls, endotheliopathy, acceleration of atherosclerosis, and post-stroke-induced decreased cell-mediated immunity [6]. The patient’s pneumonia, UTI, and bladder outlet obstruction were the leading causes of his sepsis and predisposition to massive stroke. Diagnostic and management challenges included confounding infectious symptoms that delayed recognition of evolving neurological deficits.

## Conclusions

This case highlights the rapid progression of infarction in the setting of critical ICA stenosis, infections, and delayed urinary decompression. Acute neurological patients with concurrent infections require daily neurological examinations to help reduce delayed recognition of worsening status and promote early intervention. Early recognition and coordinated multidisciplinary care can improve outcomes in similarly complex presentations. Optimizing infection control, aggressive risk factor modification, and sepsis management may reduce stroke incidence in high-risk populations. Surgical interventions can proceed only after evaluation and optimization of comorbid conditions.
